# Meeting report: ‘How do I incorporate research into my family practice?’: Reflections on experiences of and solutions for young family doctors

**DOI:** 10.4102/phcfm.v10i1.1640

**Published:** 2018-04-12

**Authors:** Kenneth Yakubu, Maria C. Colon-Gonzalez, Kyle Hoedebecke, Vasiliki Gkarmiri, Nagwa N. Hegazy, Olugbemi O. Popoola

**Affiliations:** 1 Department of Family Medicine, University of Jos and Jos University Teaching Hospital, Nigeria; 2 AfriWon Renaissance, WONCA, Young Doctor Movement for Africa; 3 UT Rio Grande Valley School of Medicine, Harlingen, United States; 4 WONCA Polaris, WONCA, Young Doctor Movement for North America; 5 Department of Family Medicine, Uniformed Services University, United States; 6 National Health Service, Primary Health Care Post of Koukkos, Pieria, Greece; 7 Family Medicine Department, Menoufia University, Egypt; 8 Al Razi, WONCA, Young Doctor Movement for Eastern Mediterranean; 9 Department of Family Medicine, Federal Medical Centre, Nigeria

## Abstract

**Background:**

Family doctors (FDs) focus on biopsychosocial components of health during consultations. However, much of the evidence employed by these doctors is produced by researchers who are not routinely involved in family practice. Family doctors competent in both clinical practice and research are essential to addressing this gap. With the growing recognition of family medicine as the specialty of choice for many young doctors, there is a scarcity of literature that describes their experiences in combining research and daily family practice.

**Aim:**

Members from Young Doctor Movements (YDMs) under the auspices of the World Organisation of Family Doctors (WONCA) sought to address this knowledge gap by reflecting on their experiences towards becoming researchers. With the assistance of senior doctors, they explored solutions that can help young FDs incorporate research into their family practice.

**Methods:**

Following an online YDM meeting, a summary of the experiences of young FDs as well as strategies useful for incorporating research into their everyday practice as FDs was prepared.

**Result:**

Nine thematic areas were derived, including experiences and motivation towards regular research, culture and environment of practice, relevance and gains of research, teamwork and mentorship.

**Conclusion:**

Family practices can incorporate research by promoting a personal and organisational research culture, highlighting gains and relevance of making it part of the profession and fostering teamwork, supportive networks and mentorship while making it enjoyable.

## Introduction

Primary care physicians competent in both clinical practice and research are needed to advance evidence-based practice, quality of care and patient safety in primary care settings.^1^ Active participation from clinicians in primary care research can close the current gap of non-clinicians producing much of the scientific evidence used in the treatment of primary care patients (who often have multi-morbidities).^2^ Primary care-based research provides a plethora of evidence applicable to everyday practice in family medicine (FM).^3^ It can also elucidate issues relevant to the undifferentiated patient, who seeks care from the family doctor (FD).^4^ Evidence pertinent to the primary care setting must achieve a person, family and community focus useful for population health and policy development.

The World Organisation of Family Doctors (WONCA) endorses seven Young Doctor Movements (YDMs) that serve as advocates for FM globally among trainees and new FDs (less than or equal to 5 years post-certification).^5^ These YDMs provide avenues for members to participate in research projects and have access to training and mentorship opportunities.^6^ These opportunities are particularly relevant, as peer mentorship has proven to be effective in improving scholarly culture as well as the overall quality of research production.^7,8^ The collaborations within these YDMs also allows participants in remote or isolated locations to engage with peers and senior colleagues who have similar interests.^9^ Expanding research competencies and techniques throughout the entirety of the global young FD community can help many learners engage in more research while improving the care of their patients.

It is not known to what extent doctors globally can demonstrate competence in both clinical practice and research. However, for the trainee and junior doctor dealing with the demands of learning and clinical practice, the combination often proves difficult because of the inherent investments needed for successful research activities.^7,10^ Some of the challenges faced include lack of time, adequate mentors and knowledge or skills in research methodologies.^7,11,12,13^ One of the most significant barriers for researchers in primary care, compared to other specialists, is the lack of recognition and promotion of their scientific productivity which is particularly important within academic settings.^13^ Some suggested answers to these challenges are flexible working hours to promote more involvement in research, remuneration per research output,^3,8^ longitudinal mentoring relationships in the areas of interest and expertise,^4,11,13,14^ personal and professional support from the academic departments and developing time management skills.^7,10,14^

In response to the paucity of scientific research produced by primary care physicians, institutions have created fellowship programmes to help clinicians gain research skills.^2^ Hard work, determination, personal resources, interest and mentors are also factors that can contribute to a medical trainee’s or junior physician’s successful career in clinical research.^10,11^ Apart from the solutions cited above, there is evidence to support the integration of research activities into undergraduate and graduate education as a means of preparing future clinicians to become primary investigators and leaders in patient-centred evidence.^7,15^ This training can result in producing effective bridges between research and clinical practice.^15^

However, considering that research in FM is still poorly developed in many countries,^16,17^ and there exists a paucity of literature on the experiences of young FDs in their journey towards becoming researchers, we provide a summary of proceedings from an international panel of young FDs, who reflected on their journey towards becoming researchers and discussed strategies that might help young FDs incorporate research into routine family practice.

## Methodology

As part of regular inter-YDM activities, a Google Hangout panel discussion about ‘the young family physician and research’ was held on 11 February 2016. The event was advertised to all the YDMs using social media, and participation was voluntary.

The lead author moderated the discussion, beginning with a brief description of the purpose of the meeting, procedures and topics for discussion. The main aim of this meeting was to reflect on the experiences of young FDs in their journey towards merging family practice and regular research. Sub-topics included participants’ description of their experience with research, what made them continue (or discontinue) a research practice on an ongoing basis, challenges they had faced and techniques used to overcome these and their recommendations to other young FDs. Senior FDs were a part of the discussions through the comments section of Google Hangout. The discussion was in English and lasted for 1 h. Even though there was a moderator, invitations for comments were open-ended, and participants were free to pursue their priorities in the discussion. Clarifications and summaries were made at regular intervals and at the end of the discussion by the moderator, who ensured that everyone participated.

Considering its relevance to promoting a culture of research and clinical practice among young FDs, the authors agreed to be part of a task group with the responsibility of producing a report from this event. The recorded discussion was then transcribed verbatim and the data were uploaded into a Google document. Authors who were part of the main event read through the transcribed interview to ensure its accuracy and completeness. The transcript was divided into six sections, and the framework approach to qualitative analysis was used to produce summaries of the discussion.^18^ The authors took time to read and familiarise themselves with a chosen section of the interview following which they derived codes inductively, keeping the meeting’s main aim in mind. They also read the other parts and had the opportunity to refine their codes. An index of all the codes was made (thematic index) and written on the margins of the transcribed text against each identified unit of meaning (indexing). The authors then discussed among themselves until they agreed with all the derived codes. They then proceeded to scan through the index as well as the main text to see if any code had been left out, or if existing ones could be better expressed. Similar codes and their representative quotes were grouped (charting), and the authors worked together to elicit an interpretation of the grouped codes resulting in overarching themes. These resulted in thematic areas aimed at describing the young FDs’ journey towards being involved in research projects and strategies to integrate research into their family practice.

## Results

Nine representatives from three YDMs including Al Razi (East Mediterranean region), WONCA Polaris (North America) and AfriWon Renaissance (sub-Saharan Africa) took part in the event. The discussion panel comprised eight young FDs and a fifth-year FM trainee (three of the young FDs had published at least five peer-reviewed articles, while five of the young FDs and the fifth-year FM trainee had published fewer than 5 articles). All the panel participants were actively involved in clinical family practice.

Two senior doctors (greater than 5 years post-qualification) with some experience in working with young FDs and 13 other young FDs including representatives from Waynakay (South America) and Vasco da Gama (Europe) participated in the discussions by providing feedback on the comments section of Google Hangout. The complete profile of young FDs who attended through the comments section was not collected during the panel discussion. However, most were part of a family practice and had shown interest in research, while the senior FDs had more than 10 publications and had been part of FM residency training in their settings.

**FIGURE 1:**
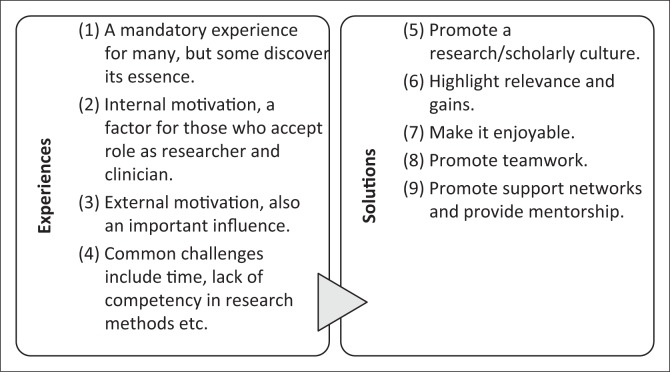
Research and family practice: A concept chart of the experiences of young FDs and proposed solutions.

Nine thematic areas were derived for young FDs already involved in clinical family practice (Figure 1). Items 1–4 describe their experiences towards being involved in research. Items 5–9 are recommended strategies which may help the young FDs figure out how to incorporate research into their family practice.

### Experiences

#### Pathway to becoming a researcher and a clinician

Most of the participants remarked that for them research began as an obligation, something required for certification or career progression. However, some of the young doctors and trainees experienced the essence of research beyond mere obligation. In addition to enriching their clinical practice, pursuing research led them along the path of self-discovery. Some representative quotes include the following:

‘I would say the majority of people [*young family physicians*] do research because it is mandatory and then they don’t do it anymore.’ (Participant 2, male, young FD)

‘It [*the experience with research*] started first as an obligation, and then I began to discover what’s important to me.’ (Participant 2, male, young FD)

#### Internal motivation towards becoming a researcher and a clinician

Participants also stated that internal motivation was a crucial factor in the journey towards regular involvement in research. A desire to pursue a personal interest, make an impact through a rich clinical experience and disseminate evidence-based practice findings specific to their settings influenced some of these young FDs to incorporate research into their clinical practice. Those who enjoyed research and thought of it as fun had a more favourable disposition towards a blended career in research and clinical practice. Some representative quotes include the following:

‘[*referring to a family practice*] … because there are cultural and background differences, what suits you in Nigeria may not suit me here in Egypt, so that motivates us to search to find the right hypothesis to research on.’ (Participant 4, female, young FD)

‘… almost all got involved in research as it was a must in their training, but when actually working on their little projects, they discover that research can actually be ‘fun’ to do, especially when it covers a topic of personal interest.’ (Participant 10, female, senior FD)

#### External motivation towards becoming a researcher and a clinician

Participants considered a scholarly environment and support networks as strong motivators towards becoming a researcher and clinician. Frequent participation in research projects helped some of the young FDs acquire more knowledge about a specific area of medicine and gain recognition as experts. Some participants said:

‘It’s a big mix of things that have to be available and come together to really make an environment that is for research and allows research to flourish.’ (Participant 2, male, young FD)

‘What I’ve really liked about it [research] is, rather than take me away from colleagues and my support network, it’s actually grown that support network.’ (Participant 2, male, young FD)

‘… that [*research*] should help you harness and define yourself as an expert in an area because as you do that research, not only are you sharing knowledge with others, you are also building your own knowledge in a specific area which then you can share with others.’ (Participant 6, female, young FD)

#### Challenges for incorporating research into family practice

Participants identified inadequate funding, lack of time and competency in research, competing family demands and difficulty in achieving a healthy work–life balance as factors hindering the incorporation of research into their family practice. Poor perception of the gains of research and a lack of interest in research were contributory factors. Previous negative experience in research, especially during the training period, was also a reason why some young FDs never embarked on the journey towards incorporating research into their family practice after the residency training. Some relevant quotes include the following:

‘If I spend one extra hour doing a research, how is that going to put anything more in my pocket? That’s the truth in this area of the world. So it’s like “why not I go and do a surgery somewhere and get some extra cash than sit down doing something?” People don’t see any long-term gains … .’ (Participant 9, male, young FD)

‘… I’m still confused as to the statistical part of the research – it’s quite challenging. I have an interesting topic I want to do a research about, but I end up being stuck along the way. That has been my experience so far.’ (Participant 7, female, young FD)

### Proposed solutions

#### Promoting a research culture and scholarly environment

Participants observed that a scholarly environment is essential for influencing young practising FDs towards becoming researchers. This change would need a shift in culture at a personal level, organisational level or both. A point system that tracks and encourages young FDs’ involvement in research and routine journal clubs can help incorporate research into one’s family practice. This requires the young FDs to be innovative while finding a healthy work–life balance. Relevant quotes include the following:

‘A culture of research is very important to have and that’s not at a country level; that’s at a location; a residency, a hospital, a training center … .’ (Participant 2, male, young FD)

‘Things like having a monthly journal club for example, having a residency director or the chief who also does research and presents is important so he’s not just saying “do it” but is also doing it. Having a point system.’ (Participant 2, male, young FD)

#### Highlighting relevance and gains

Participants felt that pointing out the relevance and impact of research to medical practice can be a way of making research more attractive to the emerging FDs. To incorporate research into one’s family practice, young FDs need to see how a regular practice of research can promote the discipline of FM and improve the status of FDs in the world of medicine. They also observed that highlighting the gains of research such as increasing camaraderie at the workplace, the ability to serve, the thrilling sense of discovery and possible financial profits may help emerging FDs realise reasons to improve their competencies in research and incorporate it into their practice. Relevant quotes include the following:

‘[*referring to the gains*] we’ve been able to really work together to not only improve family medicine, but I think really improve global health through the work we’ve been doing over the last couple of years.’ (Participant 2, male, young FD)

‘The camaraderie that we share when we work together – that sense of discovery that you are contributing to the society, the ability to serve.’ (Participant 1, male, young FD)

‘Your work appears to be without monetary rewards at first, but building up your CV with varied research, presentations and international collaboration makes you worth more to a host of potential employers. … you gain much more than just another publication. You gain knowledge of other cultures, their problems, and their solutions, and let’s not forget forging great friendships!’ (Participant 11, male, senior FD)

#### Making it enjoyable

Participants stated that making research enjoyable is key to getting younger FDs to choose the researcher’s path. Often, clinical work takes its toll on young FDs, increasing the risk for burnout. When they have fun and address their personal interests and passions, research projects can be a welcome break for young FDs.

‘I think you can make it a regular part of your life and make it enjoyable. First, you engage in projects you’re interested in.’ (Participant 6, female, young FD)

‘A number of times, I tell my residents and trainees to look beyond the exams. I tell them you’re going to pass the exams eventually, but hey, make this fun, think about making your prints.’ (Participant 1, male, young FD)

#### Promoting teamwork

Participants thought that forming dedicated research teams with people who have the same scholarly interest within a family practice can help young FDs take up their roles as researchers and clinicians. These groups would need to demonstrate a passion for research and have enthusiastic leadership. Setting clear goals and deadlines, allocating tasks and knowing each other’s limits are essential for productive group work. Turning research projects into team competition within a family practice can make it easier to incorporate research into routine clinical work. Social media can also be used to connect young FDs across the world and promote collaboration. The social interaction that results from this can make research projects a fun event and a refreshing part of clinical practice.

‘So we made teams and each team basically may add up their points and see who would have the most points. So, turning things into a competition especially for type-A personalities like most of us, always tends to spice things up a little bit and make it more fun for us in the end.’ (Participant 2, male, young FD)

‘I think that also working with people that in a sense, you know that your work personality or your work drive and ethical values are the same, also helps. I think that also knowing what are your limits, what are the deadlines and expectations [for the team work can be helpful]; so that you know how best to contribute to the project and not lose everybody’s time during the process.’ (Participant 6, female, young FD)

‘… so you can do your own group, your own Facebook group or WhatsApp group or whatever, and you start searching what you want to do.’ (Participant 4, female, young FD)

#### Promote support networks and provide mentorship

Participants thought that having multiple support networks not limited to researchers, but including the family, peers and senior colleagues, can help the young FDs keep a healthy work–life balance, making it easier to adopt research into their family practice. Mentorship (including peer mentorship) is crucial for attracting and keeping young FDs on the path towards becoming researchers.

‘Once you have this company to push you forward and are ready to help at any moment, you will feel that you want to do more.’ (Participant 4, female, young FD)

‘I definitely have to thank my wife who supports me, because of her, I’m able to do a lot of these researches well.’ (Participant 2, male, young FD)

‘I think that it’s about finding fit mentors that can guide you through the process and that are not so much focused on doing the project right, or the very thought of having the manuscript, and in the process, they’ll also teach you methodology and the things related to the research.’ (Participant 6, female, young FD)

## Discussion

For a general population of junior doctors, previous authors have suggested some strategies towards incorporating research into clinical practice. These include ensuring flexible work hours to promote more involvement in research, remuneration per research activity, longitudinal mentoring relationships in the areas of interest and expertise,^11,13,14^ personal and professional support from the academic departments and developing time management skills.^14^ All these are consistent with thematic areas 5 and 9.

While the studies cited above have focused more on the external environment of the junior doctor, discussions from this meeting have provided some unique insights. The first thematic area is evidence that young FDs understand that they are expected to be part of research activities. This suggests the outcome of efforts to promote academic FM. However, making this a mandatory part of young FDs’ training, without them taking personal ownership of the initiative, and seeing its relevance and benefits beyond certification as an FD, can be a barrier rather than a facilitator of a regular research practice following qualification. Our discussion revealed that mandatory participation in research projects might be the first experience for many young FDs. However, a process of self-discovery and self-realisation for the young FDs can occur. This can provide a sense of purpose for the young FDs, making participation in research projects much more than an academic or professional activity.

For some young FDs, the FM training may have focused on clinical tasks and responsibilities alone, with little focus on their role as researchers. Young FDs need help in achieving self-discovery during and after the FM training. Where this is not yet the norm, mentors can use research projects to explore personal passions and interests, while remaining within the overarching values of FM. Investing in providing knowledge of research methods and protected time^11,13,14^ may also be useful in helping young FDs combine research with an active clinical practice.

Material gains are essential for meeting basic human needs; however, according to Herzberg’s theory of job satisfaction, they may be described as hygiene factors and not primary motivators for a regular research and clinical practice.^19^ Our discussion highlighted non-material gains from incorporating research into family practice, including a sense of camaraderie, the thrill of discovery, being part of something relevant to the larger society and contributing to the development of the FM specialty. Making research fun and appealing to the young FDs was also identified as a way of helping young FDs incorporate research into their family practice. Previous studies have shown that gamifying work processes improves productivity and staff well-being.^20^ This may be applicable to research projects as well.

Even though this meeting established that incorporating research into family practice depends on the young FDs’ internal motivation and hard work, the thematic areas also addressed teamwork at a family practice and how it (including digital communities of practice) can help in this regard. Social learning theory, even within a digital community of young FDs, can account for learned behaviour.^21^ Indeed, when managed positively, this can help young FDs take up research projects as a regular part of their practice.

Lastly, apart from the work environment and peer influence, the thematic areas from this meeting point to less official sources of support for research – the young FD’s family. The family is the closest social unit for everyone; therefore, it can provide support and reinforce adoption and maintenance of desired behaviour.^22^

While Internet connection hindered clear communication for some parts of the meeting, the authors who were part of the event were able to fill in missing data following transcription of the discussion. The thematic areas derived from this group discussion may not be a global reflection of the experiences and opinions of all young FDs; however, the panel was diverse in its composition and its members shared similar perceptions. Our panel members may have contained more of those who were motivated towards research. Hence, the experiences of those from a different population may have been omitted.

## Conclusion

The practice of research can be a daunting challenge for emerging FDs inundated with a barrage of questions regarding what, who or how to establish themselves in everyday clinical practice. These thematic areas highlight common pathways to becoming a researcher and provide useful strategies for those interested in a blended career of research and clinical practice.
